# Mental health essentials for future healthcare professionals. A public health capacity building initiative

**DOI:** 10.1192/j.eurpsy.2021.1256

**Published:** 2021-08-13

**Authors:** M. Borrell Arrasa, M. Eissa, O. El Omrani

**Affiliations:** 1 Facultat De Medicina. Unitat Docent De Sant Pau, Universitat Autònoma de Barcelona, Barcelona, Spain; 2 Faculty Of Medicine, University Of Alexandria, Alexandria, Egypt; 3 Faculty Of Medicine, Ain Shams University, Cairo, Egypt

**Keywords:** essentials, students, mental health, capacity building

## Abstract

**Introduction:**

Mental disorders in Europe represent the leading cause of disability and the third leading cause of overall disease burden, following cardiovascular disease and cancers. As future healthcare professionals, with an imminent role in tackling this global health issue, we acknowledge that Mental Health is not adequately included in the medical curriculum. To address this gap, this workshop was created to equip medical students with the knowledge and skills that will empower them to lead a pioneering role in advocating for mental health for their patients, peers, and communities.

**Objectives:**

By the end of the workshop, all participants should have: - Increased their knowledge in the field of Mental Health disorders - Increased their skills in the field of Mental Health (preventive practices, intervention strategies towards others, etc.) - Explored the relations between stigma, vulnerability, and Mental Health - Strengthened the international perspective and global understanding concerning mental health issues

**Methods:**

This workshop utilized principles of non-formal education and was planned according to the 4MAT system to meet all four learning styles through theory blocks, space for reflection, practical application of knowledge, and future opportunities for application of content. This was achieved through various methodologies including presentations, self-discovery activities and exercises, roundtable debates, simulations, and role-playing.

**Results:**

obtained

**Figure:**
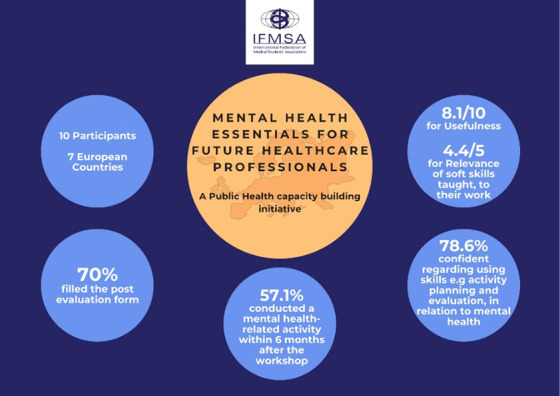

**Conclusions:**

This workshop highlighted the importance of building the capacity of medical students to tackle the burden of Mental Health globally and within the region, and how similar student-led initiatives can further empower them to be change agents and impactful advocates for better Mental Health in their own communities.

